# Emergence of serotype 19A *Streptococcus pneumoniae* after PCV10 associated with a ST320 in adult population, in Porto Alegre, Brazil

**DOI:** 10.1017/S0950268819000013

**Published:** 2019-02-22

**Authors:** M.P. Mott, J. Caierão, G.R. Cunha, M.M. Del Maschi, K. Pizzutti, P. d'Azevedo, C.A.G. Dias

**Affiliations:** 1 Basic Health Department, Federal University of Health Sciences of Porto Alegre, Porto Alegre, Brazil; 2 PROADI-SUS Project Department, Moinhos de Vento Hospital, Porto Alegre, Brazil

**Keywords:** Brazil, serotype 19A, *Streptococcus pneumoniae*, ST320

## Abstract

Use of pneumococcal conjugate vaccines has caused emergence of non-vaccine serotypes. No Brazilian data specifically about serotype 19A are available. We aimed to evaluate the frequency of occurrence, susceptibility profile and molecular epidemiology of serotype 19A before and after vaccine introduction in Brazil. Pneumococcal identification was performed by the conventional method. Strain serotype was determined by multiplex polymerase chain reaction (PCR) and/or Quellung reaction. Resistance was determined by Etest^®^ and PCR was performed to determine the presence of macrolide resistance genes, *erm*B and/or *mef*A. Pneumococci were typed by Multilocus Sequence Typing. Thirty-eight serotype 19A *Streptococcus pneumoniae* were recovered, mostly from invasive diseases. Prevalence of serotype 19A increased following vaccination (from 3.5% before vaccination to 8.1% after, *p* = 0.04196). Non-susceptibility increased to most antimicrobials after vaccine introduction and was associated with clonal complex (CC)320. MLST showed nine different STs, which were grouped in one main CC: CC320 (63.9%). During the post-vaccination era, the frequency of this serotype increased significantly from 1.2% in 2011 to 18.5% in 2014 (*p* = 0.00001), with a concomitant decrease in the genetic variability: ST320 consistently predominated after vaccine-introduction (61.1%). Overall, our results showed a post-PCV10 increase in the frequency of serotype 19A. This was accompanied by a selection of CC320 and antimicrobial resistance.

## Introduction

Infections caused by *Streptococcus pneumoniae* are a public health problem worldwide, notably in developing countries, where there are high rates of mortality and morbidity [1, 2]. In order to reduce the occurrence of pneumococcal diseases, as well as the dissemination of non-susceptible to penicillin and/or multidrug-resistant isolates, vaccination is a powerful tool [3, 4].

In the year 2000, a conjugate 7-valent polysaccharide formula (PCV7) was introduced, targeting the most frequent serotypes causing infectious diseases (4, 6B, 9 V, 14, 18C, 19F, and 23F). Thereafter an increased prevalence in non-PCV7 serotypes was observed in invasive pneumococcal disease (IPD) [4–9]. Serotype 19A and in particular genotype ST320 [9], has been associated with higher resistance rates, including penicillin non-susceptibility and multidrug-resistance [7–10].

In order to increase the protection and to control the worrisome emergence of this serotype, the 13-valent (PCV13) formula (adding serotypes 1, 3, 5, 6A, 7F and 19A) was implemented for use in the USA [11]. In Brazil, however, only the 10-valent vaccine (PCV10) is licensed for use in the public system and does not target serotype 19A. The positive impact of PCV10 has been clearly observed, with a decrease in the incidence of vaccine-targeted serotype-specific IPD, as well as child mortality rates and pneumonia hospitalisation [12–16]. On the other hand, the emergence of non-PCV10 serotypes – including 19A – has been recognised [6, 8, 9, 11, 14, 17].

To date, no studies focusing on serotype 19A after PCV10 introduction in Brazil have yet been published. Therefore, in the current study we analyse the frequency of occurrence, susceptibility profile and molecular epidemiology of serotype 19A *S. pneumoniae* from the pre- and post-vaccine periods in Porto Alegre, South Brazil.

## Methods

*S. pneumoniae* serotype 19A obtained from patients with invasive and non-invasive diseases from 2008 to 2014 were included in this study. Isolates recovered from 2008 to 2010 were of the pre-vaccine period, while the ones recovered from 2011 to 2014 were the post-vaccine period isolates. These isolates were obtained from three hospitals in Porto Alegre (metropolitan area with more than 4 million inhabitants), South Brazil: Grupo Hospitalar Conceição (GHC), Hospital Mãe de Deus (HMD) and Hospital de Clínicas de Porto Alegre (HCPA). Each hospital's Research Ethics Committee approved the study. Identification of isolates was performed by conventional methods: Gram stain, optochin susceptibility and bile solubility [18].

The DNA extraction for subsequent experiments was performed following the Centres for Disease Control and Prevention (CDC) recommendations: one loop of a fresh growth of *S. pneumoniae* was suspended in 200 µl of 5% Chelex^®^ resin solution (Bio-Rad, Hercules, CA, USA) containing 200 µg/ml of Proteinase K (Invitrogen, Life Technologies, Carlsbad, CA, USA) [19]. The suspension was incubated at 56 °C for 1 hour and at 95 °C for 10 min. The process was concluded by centrifugation at 12.000 rpm for 3 min and the supernatant was stored at −20 °C [19]. Isolates were serotyped using the sequential multiplex PCR developed by Dias *et al*., [20], for the most common serotypes reported in Latin America and/or the Quellung reaction [21].

Minimal Inhibitory Concentrations (MICs) to penicillin, ceftriaxone, meropenem, tetracycline, erythromycin, trimethoprim-sulfamethoxazole, levofloxacin and vancomycin were determined using Etest^®^ strips, following the manufacturer's instructions and interpreted according to breakpoints recommended by the Clinical and Laboratory Standards Institute [22]. The reference strain *S. pneumoniae* ATCC 49619 was included for quality control purposes. Isolates non-susceptible to erythromycin were submitted to duplex PCR reaction for the detection *erm*B and *mef*A genes [23].

Molecular typing of serotype 19A was performed using Multilocus Sequence Typing (MLST) [24]. Allele profiles and sequence types (ST) were obtained from the MLST database (http://pubmlst.org/spneumoniae/) [25]. Bionumerics software 6.6 (http://www.applied-maths.com/knowledge-base/citing-applied-maths-products) (Applied Maths, Sint-Martens- Latem, Belgium) was used to analyse from the MLST data and construct a Minimum Spanning Tree (MST). This method of clustering calculates a similarity matrix based on a categorical coefficient, since the allele numbers are arbitrary. All STs assigned to the same group must share all identical alleles (seven/seven). For clonal complexes (CCs), a cut-off point of at least five identical loci was used.

Statistical analysis was performed using SPSS 19 software by chi-square test and Fisher's exact test whenever necessary. Results were considered statistically significant when *p* < 0.05.

## Results

**Fig. 1.**
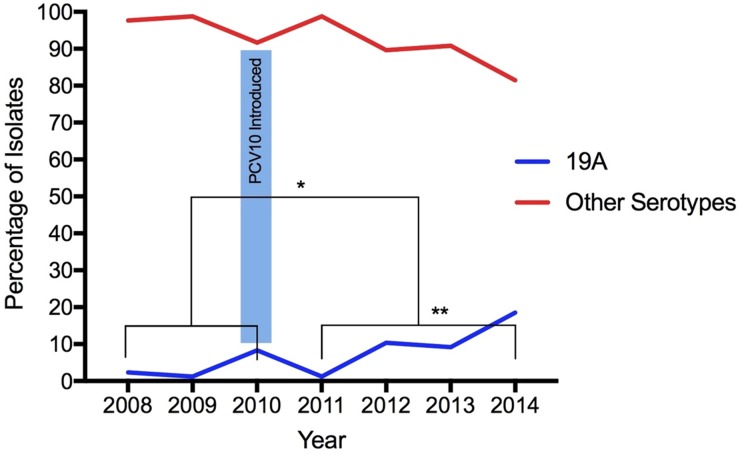
Increase of *Streptococcus pneumoniae* serotype 19A isolates after the introduction of PCV10. (*) Statistically significant (*p* = 0.04196) comparing pre- and post-vaccination; (**) statistically significant (*p* = 0.00001) comparing only post-vaccine period 2011 with 2014.

From 2008 to 2014, 568 *S. pneumoniae* isolates were serotyped, 30.5% (173/568) recovered during the pre-vaccine period and 69.5% (395/568) recovered during the post-vaccination period. According to age, 9% (51/568) of isolates were obtained from young children (under 5 years of age), 5.3% (30/568) from children (aged 5 to 18 years), 55.8% (317/568) from adults (aged 19–64 years) and 26% (148/568) from elderly (65 years old and above). The age of 22 individuals was not available. Isolates obtained from IPD were the most frequent origin of pneumococci, representing 74.3% (422/568), whereas 17 pneumococci were from unknown specimens. Overall, 6.7%, (38/568) were serotyped as 19A: 3.5% (6/173) before and 8.1% (32/395) after vaccine introduction, demonstrating a statistically significant increase in the frequency of this serotype (*p* = 0.04196) (Fig. 1). The age of patients infected with serotype 19A pneumococci ranged from 1 to 80 years old (average: 46.5 years old; median: 54 years old). Only 13.1% patients were 5 years old or less (Table 1).

**Table 1.** Characteristics 38 *S. pneumoniae* isolates of serotype 19A recovered during pre- and post-vaccine period

Period	Internal identification of the isolate	Specimen	Age of patients (years)	ST	PEN (μg\ml)	CRO (μg\ml)	MER (μg\ml)	TET (μg\ml)	SXT (μg\ml)	ERY (μg\ml)	*erm*B	*mef*A
Pre-Vaccination												
	042-09	B	37	4967	0.25	0.06	⩽0.06	0.5	>4	⩽0.03	–	–
	022-10	B	21	8800	⩽0.03	⩽0.03	⩽0.06	⩽0.25	0.25	⩽0.03	–	–
	025-11	B	62	199	0.12	0.06	⩽0.06	⩽0.25	⩽0.12	⩽0.03	–	–
	015-08	PF	3	2878	0.12	⩽0.03	⩽0.06	⩽0.25	1	0.06	–	–
	055-11	B	38	2878	0.12	⩽0.03	⩽0.06	⩽0.25	0.5	⩽0.03	–	–
	015-10	–	–	–	–	–	–	–	–	–	–	–
Post-Vaccination												
	030-12	B	19	2878	0.12	0.06	⩽0.06	⩽0.25	0.5	0.06	–	–
	062-12	B	1	2878	0.094	0.06	⩽0.06	⩽0.25	0.25	⩽0.03	–	–
	046-13	TA	–	199	⩽0.03	⩽0.03	⩽0.06	⩽0.25	0.064	0.047	–	–
	022-12	B	49	733	⩽0.03	⩽0.03	⩽0.06	⩽0.25	0.75	⩽0.03	–	–
	001-14	B	33	733	0.016	⩽0.03	⩽0.06	⩽0.25	3	⩽0.03	–	–
	049-14	B	61	733	0.016	⩽0.03	⩽0.06	⩽0.25	1.5	⩽0.03	–	–
	060-14	B	69	2260	0.064	⩽0.03	⩽0.06	⩽0.25	1.5	0.047	–	–
	014-14	B	72	9678	0.047	0.06	⩽0.06	⩽0.25	0.25	⩽0.03	–	–
	130-12	CSF	79	8884	4	2	1	>8	>4	>32	pos	pos
	167-11	B	67	320	2	2	1	>8	>4	>32	pos	pos
	006-12	S	38	320	4	2	1	⩽0.25	>4	4	neg	pos
	025-12	B	67	320	4	2	1	>8	>4	>32	pos	pos
	054-12	S	80	320	4	1	0.38	>8	3	>32	pos	pos
	010-13	PF	4	320	1.5	2	0.5	>8	>4	>32	pos	pos
	063-13	B	75	320	2	1.5	0.5	>8	>4	>32	pos	pos
	112-13	PF	2	320	4	1	0.25	4	>4	>32	pos	pos
	115-13	B	47	320	1.5	1.5	0.38	⩽0.25	2	2	neg	pos
	016-14	B	50	320	0.75	1	0.25	⩽0.25	3	3	neg	pos
	017-14	B	78	320	0.75	1	0.38	3	3	>32	pos	pos
	030-14	B	60	320	2	2	0.38	⩽0.25	1.5	2	neg	pos
	037-14	B	57	320	3	2	0.25	⩽0.25	1	0.75	neg	pos
	056-14	B	57	320	0.75	0.75	0.25	⩽0.25	1.5	2	neg	pos
	057-14	CSF	57	320	0.75	0.75	0.25	⩽0.25	1.5	2	neg	pos
	065-14	B	1	320	2	2	0.38	1.5	1	>32	pos	pos
	024-14	CSF	30	320	1.5	1.5	0.38	3	0.75	>32	pos	pos
	025-14	B	30	320	1.5	2	0.38	1	0.38	>32	pos	pos
	014-12	TA	59	320	4	2	1	>8	>4	>32	pos	pos
	041-14	B	63	320	1.5	2	0.25	3	1.5	>32	Pos	pos
	062-13	B	61	320	2	2	0.25	3	3	>32	pos	pos
	092-14	B	41	320	0.125	0.38	⩽0.06	6	1.5	>32	pos	pos
	111-13	BAL	54	320	–	–	–	–	–	–	–	–
	087-12	S	54	–	–	–	–	2	0.5	12	neg	pos

ST, sequence type; B, blood; BAL, broncho alveolar lavage; CSF, cerebrospinal fluid; PF, pleural fluid; TA, tracheal aspirate; S, sputum. PEN, penicillin; CRO, ceftriaxone; MER, meropenem; TET, tetracycline; SXT, trimethoprim-sulfamethoxazole; ERY, erythromycin. Neg, negative; pos, positive.

MIC penicillin parenteral (nonmeningitis): ⩽2.0–4.0 – ⩾8.0 mcg/ml; MIC penicillin parenteral (meningitis): ⩽0.06–⩾0.12 mcg/ml; MIC ceftriaxone (nonmeningitis): ⩽1.0–2.0 – ⩾4.0 mcg/ml; MIC ceftriaxone (meningitis): ⩽0.5 −1.0 – ⩾ 2.0 mcg/ml; MIC meropenem: ⩽0.25–0.50 – ⩾1.0 mcg/ml; MIC tetracycline: ⩽1.0–2.0 – ⩾4.0 mcg/ml; MIC trimethoprim-sulfamethoxazole: ⩽0.5–1/19-2/38 – ⩾ 4/76 mcg/ml. MIC erythromycin: ⩽0.25–0.50 – ⩾1.0 mcg/ml.

Considering the post-vaccine period, the proportion of serotype 19A increased year-by-year: in 2011 (1.2%, 2/161), 2012 (10.4%, 8/77), 2013 (9.2%, 7/76) and 2014 (18.5%, 15/81). Comparing 2011 with 2014, the frequency of this serotype increased significantly (*p* = 0.00001) (Fig. 1).

**Fig. 2.**
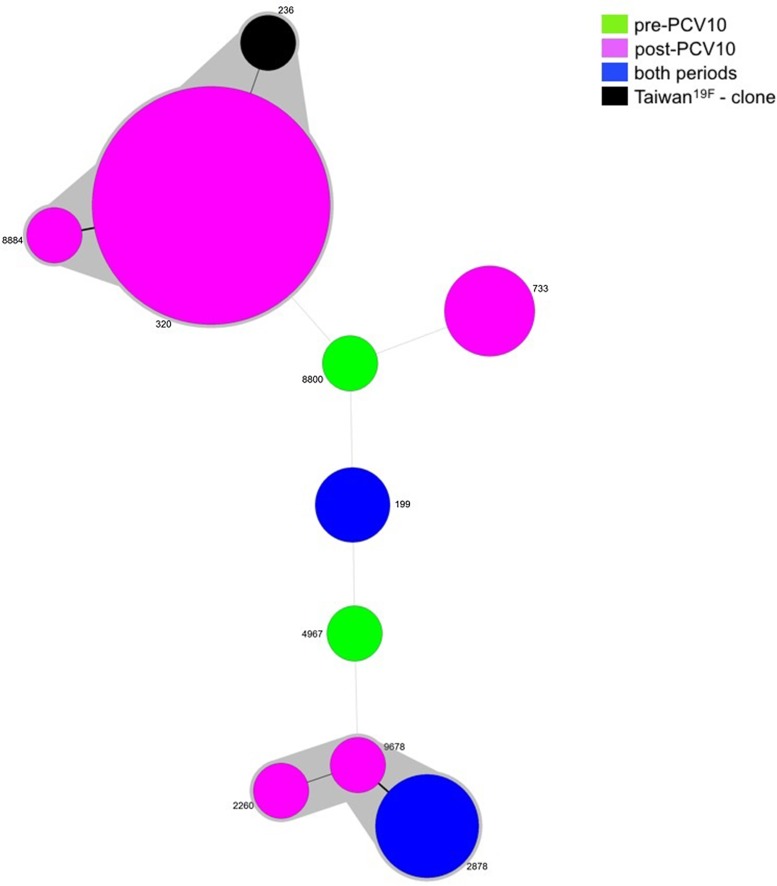
Minimum spanning tree (MST) obtained from MLST results. Size of the circles is proportional to the number of isolates. The traces connecting the circles show the distance between every genotype in a number of loci. Every genotype contained in the same gray shadow is considered as a unique clonal complex (CC).

Since two isolates were not viable, MLST was performed for 36 isolates belonging to serotype 19A, five pre- and 31 post-vaccine. Nine different STs were obtained and most of the isolates grouped into one main CC: CC320 (23/36, 63.9%), in which ST320 and ST8884 were identified. ST320 is double locus variant (DLV) of ST236, which is the ST of the PMEN clone Taiwan^19F^-14, whereas ST8884 is a single locus variant (SLV) of ST320. Another CC was observed including isolates belonging to ST2878 (4/36, 11.1%), ST2260 (1/36, 2.8%) and ST9678 (1/36, 2.8%). The remaining isolates represented singletons (Table 2 and Fig. 2).

**Table 2.** Results from MLST analysis of 36 serotype 19A *S. pneumoniae* isolates.

ST	N (%)	Pre (%)/Post (%)
320	22 (61.1%)	0/22 (100%)
8884	1 (2.8%)	0/1 (100%)
2260	1 (2.8%)	0/1 (100%)
2878	4 (11.1%)	2 (50%)/2 (50%)
9678	1 (2.88%)	0/1 (100%)
733	3 (8.3%)	0/3 (100%)
199	2 (5.5%)	1 (50%)/ 1 (50%)
4967	1 (2.8%)	1 (100%)/0
8800	1 (2.8%)	1 (100%)/0

ST, sequence type; N, number of isolates; Pre, pre-vaccination period; Post, post-vaccination period.

While ST199 and ST2878 were recovered from both the pre- and post-vaccine period, ST8800 and ST4967 were found exclusively prior to vaccine introduction. On the other hand, isolates belonging to CC320, ST2260, ST733 and ST2260 were present only after vaccine implementation (Table 2 and Fig. 2).

According to the MLST database, most STs described among our isolates belong exclusively to serotype 19A (ST2878, ST9678, ST8800, ST2260 and ST8884). Interestingly, all but ST8800 had been previously recovered in Brazil. On the other hand, some STs are represented by more than one serotype, but 19A was the most frequent (Table 3).

**Table 3.** Sequence types observed during the study period which include *Streptococcus pneumoniae* isolates serotypes other than 19A

ST	Period	MLST database	Region
320	Post	There are 424 records, the most prevalent serotype is 19A (372/424, 87.7%), followed by serotype 19F (47/424, 11.1%), serogroup 19 (3/424, 0.7%) and serotype 6B (2/424, 0.5%).	Around the world, including Brazil
199	Pre and Post	There are 708 records, the most prevalent serotype is 19A (443/708, 62.6%), followed by 15B/C (123/708, 17%), 15C, (58/708, 8%), 15B (57/708, 8%) and other with lower frequency (3, 6, 7C, serogroup 15, 15A, 15F, serogroup 19, 19B, 19F, 21.39, 23A, 23F and without serotype).	Around the world, including Brazil
733	Post	19F and 19A There are 22 records: 19A (21/22, 95.5%) and 19F (1/22, 4.5%).	Brazil and Germany
4967	Pre	There is one record, serotype 23F	Brazil

ST, sequence type; MLST database, available: http://pubmlst.org/spneumoniae/

Penicillin MICs were determined for 35 serotype 19A isolates (five pre- and 30 post-vaccine introduction and varied from ⩽0.03 µg/ml to 4 µg/ml. MIC_50_ and MIC_90_ for all isolates of serotype 19A were 1.5 µg/ml and 4 µg/ml, respectively. However, by excluding CC320 isolates, MIC_50_ and MIC_90_ decreased to 0.094 µg/ml and 0.12 µg/ml, respectively. Ceftriaxone MICs for all isolates varied from ⩽0.03 µg/ml to 2.0 µg/ml. MIC_50_ was 1.0 µg/ml and MIC_90_, 2.0 µg/ml. Again, by excluding CC320 isolates, MIC_50_ and MIC_90_ decreased to 0.047 µg/ml and 0.064 µg/ml, respectively.

Table 4 shows the susceptibility profile of all serotype 19A isolates. All isolates were susceptible to vancomycin and levofloxacin. Considering all other antimicrobials, an increased frequency of non-susceptibility was seen in the post-vaccine period, mostly associated with CC320 (ST320 and ST 8884) (Tables 1 and 4).

**Table 4.** Antimicrobial susceptibility profile of 36 *S. pneumoniae* from serotype 19A pre- (five Isolates) and post-vaccine period (31 isolates*)

	Pre-vaccine			Post-vaccine		
	*S* (%)	*I* (%)	*R* (%)	*S* (%)	*I* (%)	*R* (%)
Penicilin (non-meningitis)^a^	5 (100)	0 (0)	0 (0)	23 (76.7)	7 (23.3)	0 (0)
Penicilin (meningitis)^a^	1 (20)	–	4 (80)	7 (23.3)	–	23 (76.7)
Ceftriaxone(non-meningitis)^a^	5 (100)	0 (0)	0 (0)	15 (50)	15 (50)	0 (0)
Ceftriaxone (meningitis)^a^	5 (100)	0 (0)	0 (0)	9 (30)	9 (30)	12 (40)
Meropenem^a^	5 (100)	0 (0)	0 (0)	16 (53.3)	9 (30)	5 (16.7)
Tetracycline	5 (100)	0 (0)	0 (0)	16 (51.6)	6 (19.4)	9 (29)
Erythromycin	5 (100)	0 (0)	0 (0)	8 (25.8)	1 (3.2)	22 (71)
Trimethoprim-Sulfamethoxazole	3 (60)	1 (20)	1 (20)	6 (19.3)	17 (54.9)	8 (25.8)

S, susceptible; I, intermediate; R, resistant.

a In the post-vaccine period one isolate was not available for Penicillin, Ceftriaxone and Meropenem.

## Discussion

Serotype 19A has been a subject of concern in some regions since PCV7 implementation, due to an increase in prevalence and antibiotic resistance [7, 8–10, 26, 27]. This is the first Brazilian study to evaluate the frequency, susceptibility profile and molecular epidemiology of this serotype before and after vaccination with PCV10.

While data from Latin America and Caribbean showed that, from 1990 to 2010, the frequency of serotype19A increased from 1.5 to 4.9% [28], a study conducted by Santos *et al*., demonstrated a stable incidence of serotype 19A among Brazilian children, soon after PCV10 implementation [13]. Also, in a previous study, our group did not detect a significant increase in the frequency of serotype 19A among adults, comparing the pre- and post-vaccination periods [29]. Studies that investigated the effect of PCV10 introduction on nasopharyngeal colonisation among Brazilian children did not observe an increase in the proportion of children colonised with isolates belonging to serotype 19A [17, 30].

On the other hand, Domingues *et al*., designed a case-control study to evaluate the effectiveness of PCV10 vaccine in Brazilian children and serotype 19A was the third most common in IPD. However, the study concluded that PCV10 might provide cross-protection against serotype 19A [14]. A study describing the epidemiology of IPD in Quebec, where PCV10 was used between 2009 and 2010, supports the idea that it can provide some protection against disease caused by serotype 19A [31]. Other countries that use PCV10, including Finland, New Zealand and Chile, also found a decrease in the numbers of cases of IPD caused by serotype 19A after the introduction of the vaccine [32].

Although, such cross-reactivity may not interfere in the carrier status of the children, thus providing conditions for transmission to adults. Apart from that, our data show that the increase of serotype 19A was consistent comparing the pre- and post-vaccine periods. It should be considered, however, that the increase in 19A is unlikely to be caused exclusively by vaccination [33, 34]. Other factors may be associated, such as, the temporal variations in the distribution of pneumococcal serotypes, independent of selective pressure by antibiotic use or vaccination [33, 34]. We should also consider that following vaccine implementation the hierarchy of pneumococci in carriage and disease was disrupted and now that some time has passed, this is settling with a more stable order being created [35]. However, we should also consider dissemination of some specific clones due to antimicrobial pressure. This was seen globally, with the spread of multidrug-resistant ST320, belonging to serotype 19A, in absence of a vaccination program, related to antibiotic pressure [36–39].

The inherent characteristics of some specific clones of serotype 19A also clearly appeared in our study, i.e., antimicrobial resistance. Indeed, molecular epidemiology analysis demonstrated a selection, post-vaccine, of CC320, considerably decreasing the genetic variability in the serotype 19A population. The association between this CC with penicillin non-susceptibility and/or multidrug-resistant isolates is well known [7–9] and our results are in accordance with this observation.

ST320 is a DLV of the worldwide-established Taiwan^19F^-14 (ST236) clone, primarily associated with serotype vaccine 19F [40]. This clone, due to a capsule switching event, exhibits serotype 19A [40]. The genetic evolution from ST236 to ST320 provided advantages associated with the transfer of penicillin-resistant genes [40]. ST320 was the most important clone that emerged after PCV7 introduction in several regions the world, mainly in the USA [7, 9, 40], becoming a subject of concern worldwide because of its consistent association with antibiotic resistance [36–38].

Interestingly, we observed some combinations of ST and serotype that may represent a capsular switching event. ST733, for example, may be a 19F-19A switch, as the first described pneumococci (2001) in Brazil of this ST belonged to serotype 19F, in the absence of vaccination. All other 21 ST733 isolates (one from Germany and 20 from Brazil) were serotyped as 19A (MLST database) [25]. Also, ST4967 was described in Brazil as serotype 23F pneumococci [25]. Our results show that this ST was recovered as 19A in an isolate from the pre-vaccine period, indicating that capsular switch occurred independent of selective pressure by the vaccine.

Higher MICs for penicillin and ceftriaxone occurred only in CC320 (ST320 and ST8884), as well as the increased proportion of multidrug-resistance. The presence of *erm*B and *mef*A genes was observed only in ST320 and ST8884 isolates. The *erm*B gene was associated with higher MICs for erythromycin. It is important to mention that a study previously performed in Brazil showed that non-susceptibility to penicillin and ceftriaxone was low (0.6 and 1.3%, respectively), being detected especially among isolates belonging to serotypes 19A and 14 [41].

Our study has some limitations. Although Porto Alegre has more than 4 million inhabitants in its metropolitan area, isolates were obtained exclusively from patients of this city, limiting a wider interpretation of the results obtained. Ideally, the inclusion of isolates obtained in other regions of Brazil would increase the external validity of the investigation.

Overall, our results indicate an increase in the frequency of serotype 19A in invasive and non-invasive isolates of *S. pneumoniae* in the post PCV10 period. This was accompanied by a selection of CC320 and, consequently, antimicrobial non-susceptibility also increased. Surveillance studies must be performed systematically to monitor the vaccine-induced changes and changes to serotype 19A and see if this trend continues.
